# Highly Sensitive Detection of the Antibiotic Ciprofloxacin by Means of Fiber Enhanced Raman Spectroscopy

**DOI:** 10.3390/molecules24244512

**Published:** 2019-12-10

**Authors:** Sebastian Wolf, Timea Frosch, Juergen Popp, Mathias W. Pletz, Torsten Frosch

**Affiliations:** 1 Leibniz Institute of Photonic Technology, 07745 Jena, Germany; 2 Institute of Physical Chemistry, Friedrich Schiller University, 07743 Jena, Germany; 3 Abbe Center of Photonics, Friedrich Schiller University, 07745 Jena, Germany; 4 Institute of Infectious Diseases and Infection Control, University Hospital, 07747 Jena, Germany

**Keywords:** Fiber enhanced Raman spectroscopy, antibiotics, ciprofloxacin, fiber sensing, ultrasensitive, therapeutic drug monitoring, sepsis, hollow core photonic crystal fiber, photonic bandgap

## Abstract

Sepsis and septic shock exhibit a rapid course and a high fatality rate. Antibiotic treatment is time-critical and precise knowledge of the antibiotic concentration during the patients’ treatment would allow individual dose adaption. Over- and underdosing will increase the antimicrobial efficacy and reduce toxicity. We demonstrated that fiber enhanced Raman spectroscopy (FERS) can be used to detect very low concentrations of ciprofloxacin in clinically relevant doses, down to 1.5 µM. Fiber enhancement was achieved in bandgap shifted photonic crystal fibers. The high linearity between the Raman signals and the drug concentrations allows a robust calibration for drug quantification. The needed sample volume was very low (0.58 µL) and an acquisition time of 30 s allowed the rapid monitoring of ciprofloxacin levels in a less invasive way than conventional techniques. These results demonstrate that FERS has a high potential for clinical in-situ monitoring of ciprofloxacin levels.

## 1. Introduction

Sepsis and septic shock are among the most alarming diseases. Every year, around 0.8 million people develop sepsis in Germany and the mortality rate is around 50% [1,2]. Since delayed or inadequate antibiotic treatment increases the mortality, fast and accurate therapy is essential for the survival of the patient [2,3]. The effective therapeutic antibiotics concentration [3,4] is often missed for patients with sepsis. Individual variations in pharmacokinetic profile of the administered drugs are highly unpredictable and dynamic. Therefore, fast point-of-care monitoring is needed for individualized dose-adaptation [5,6,7]. The respective technique should be minimally invasive. In this article, a sophisticated method based on fiber enhanced Raman spectroscopy (FERS) is presented, which shows high potential for monitoring of antibiotic kinetics. Ciprofloxacin is recommended as part of the initial antibiotic combination in respiratory, abdominal, and urogenital sepsis [4], since it has good tissue penetration and a broad spectrum against different pathogens [8,9]. The mean serum levels of ciprofloxacin were reported between 6–8 µM for newborn babies suffering from sepsis [5] and 8 to 11 µM [10] for healthy subjects. For seriously ill patients, ciprofloxacin levels around 12 µM have been documented, with a high interpersonal variance due to the patients’ individual pathophysiology [6]. The high inter-individual variation in ciprofloxacin kinetics was confirmed by a study of severely ill sepsis-patients, with additional dose-accumulation above 18 µM [7].

To access the antibiotic concentration-profile over time, blood samples have to be taken from the patients every 30 min to 1 h. After sample preparation they are routinely analyzed using high performance liquid chromatography (HPLC) coupled with UV- or mass-spectroscopy (MS) [11,12,13,14]. Although HPLC-MS is a highly sensitive technique for the measurement of ciprofloxacin (concentrations down to approximately 0.28 µM have been achieved [11]), it requires measurement times in the range of hours, depending on the procedure. These instruments are expensive, bulky, and require trained personal for their operation. Especially in the case of sepsis, where time is an extremely critical factor for the outcome of the therapy, (near) real time bedside monitoring of the antibiotic levels would strongly benefit the patient.

Raman spectroscopy is an arising technique [15,16,17,18,19,20,21,22,23], which is based on the vibrations of molecules [24,25,26,27,28] and provides high chemical selectivity [29,30,31,32]. This direct method is non-destructive and can be applied for quantitative measurements [33,34,35,36,37]. However, the weak Raman scattering has to be enhanced with elaborated techniques to achieve high sensitivity. Equation (1) provides an expression for the Raman scattering intensity.

(1) $$I_{Stokes}\propto NI_{0}{\left(\omega_{0}-\omega_{r}\right)}^{4}{\left|\alpha \right|}^{2}$$

**Figure 1:**
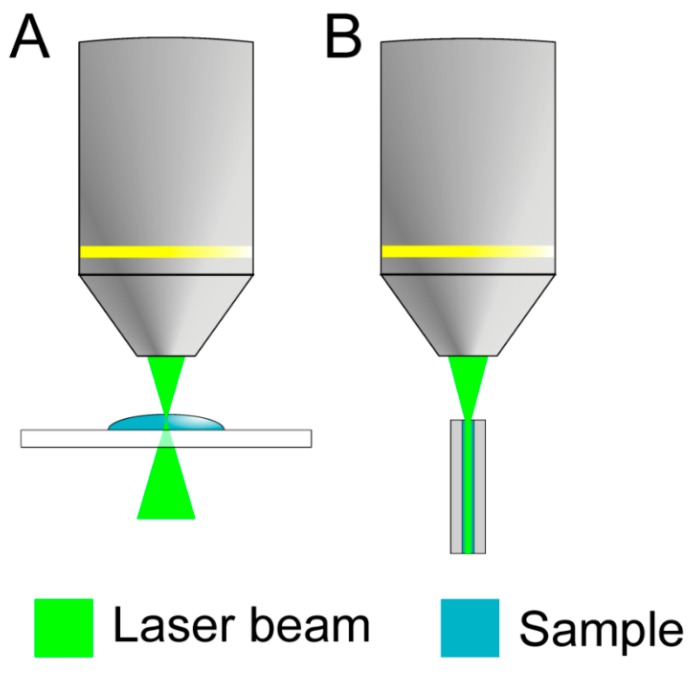
A strongly increased interaction volume is achieved in fiber enhanced Raman spectroscopy (FERS) (**B**) in comparison to a conventional confocal setup (**A**), where a tiny scattering volume of the antibiotic solution contributes to the collected Raman signal.

*N* is the number of molecules, *I*_0_ the laser intensity, ω the laser and Raman frequencies, and α is the molecule’s polarizability tensor. The Raman intensity *I_stokes_* can be increased by increasing the excitation frequency *ω*_0_, the laser power *I*_0_, or the number of molecules *N*, which contribute to the Raman signal [38]. The last approach is exploited in fiber-enhanced Raman spectroscopy (FERS), where the light is guided in the hollow fiber [38,39,40,41,42,43,44,45,46,47,48] and can strongly interact with the drug molecules over an extended interaction length [49,50,51,52,53,54] (Figure 1). Within the last decades, a lot of effort was invested in studying the fiber enhancement effect of photonic crystal fibers [55,56,57,58,59,60,61,62,63,64,65,66,67,68,69,70,71,72,73]. Here, we exploit the bandgap shift in a photonic crystal fiber into the near-infrared range, where fluorescence is strongly suppressed. The fiber features a central hollow core, with favorable light coupling properties [74].

## 2. Materials and Methods

### 2.1. Raman Spectroscopy

Ciprofloxacin hydrochloride was purchased from Santa Cruz Biotechnology and dissolved in distilled water. The background signal of the sensor fiber was acquired with pure water. After every measurement of ciprofloxacin, the fiber was thoroughly flushed and cleaned with distilled water. Each concentration of ciprofloxacin was measured three times with an integration time of 3 s and averaging over 10 spectra.

The fiber features a small core diameter (d) of 10 µm (70 µm including PCF microstructure) and only a small sample volume of 0.58 µl was needed for a fiber with length (l) of 15 cm.

To compensate for all environmental influences, such as power fluctuations, variations of the coupling efficiency, heating, or other effects, the Raman band of water at 1645 cm^−1^ was taken as internal standard. After spectral processing (multiplicative scattering correction MSC and background correction), a Lorentzian peak fitting (Levenberg-Marquardt nonlinear least squares fit with the three strongest ciprofloxacin peaks at 1389 cm^−1^, 1480 cm^−1^, and 1616 cm^−1^ (Figure 3)) was performed.

The FERS measurements were compared with conventional cuvette measurements with identical setup parameters (integration time and laser power). The noise levels for the calculation of the limit of detection (LOD) and the signal to noise ratio (SNR) were derived from the standard deviation σ of the water background (between 1200 cm^−1^ and 1600 cm^−1^) including broadband fluctuations with a sigma of 263 counts. The peak height of the Raman band of ciprofloxacin at 1389 cm^−1^ was used as signal for the SNR calculations (see Table 1).

### 2.2. Density Functional Theory Calculations

For a better understanding of the Raman bands, specifically their assignment and interpretation, vibrational modes and Raman activities were calculated with density functional theory (DFT) using Gaussian 09 [75]. The hybrid exchange correlation function with Beckes three-parameter exchange functional (B3) [76], slightly modified by Stephens et al. [77], coupled with the correlation part of the functional from Lee, Yang, and Parr (B3LYP) [78], and Dunning’s triple correlation consistent basis set of contracted Gaussian functions with polarized and diffuse functions (cc-pVTZ) [79,80] was applied. The wavenumber positions of the strongest bands in an FT-Raman spectrum of ciprofloxacin were scaled to the scattering activities of the DFT calculation. By minimizing the mean average deviation (MAD), the frequency-scaling factor (0.9935) was calculated and the intensity correction [81] was estimated.

## 3. Results and Discussion

**Figure 2:**
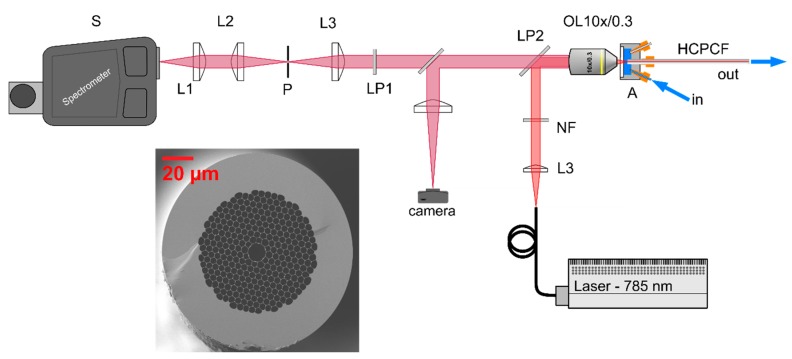
The FERS setup consists of a 785 nm excitation laser, a 10×/0.3NA microscope objective (**OL**), laser cleaning (**NF**) and long-pass filters (**LP**), a hollow core fiber (**HCPCF**), a pinhole (**P**), and several lenses (**L**). The laser beam is reflected by the long pass dichroic mirror (**LP2)** into the microscope objective and further focused into the 10 µm hollow core of the sensor fiber. Within the fiber, the light interacts with the ciprofloxacin molecules. The scattered light is collected by the objective (**OL**) and transformed into a parallel beam by the objective. Rayleigh scattering is removed with long pass filters (**LP**). The signal is further filtered with two lenses (**L2 and L3**) and a pinhole (**P**) to block the glass signal originating from the silica cladding structure of the fiber. The pinhole size matches the core image of the intermediate image between lens L2 and L3 and is thus blocking light from the glass crystal structure. A camera with flipping mirror is used to simplify the alignment process.

In fiber enhanced Raman spectroscopy (FERS), both the laser light and Raman scattered light are guided in a hollow sensor fiber (Figure 1) which is also filled with the antibiotic solution. In doing so, a strongly increased interaction volume was achieved in FERS (Figure 1B) in comparison with a conventional confocal setup, where only a tiny scattering volume of the antibiotic solution contributes to the collected Raman signal (Figure 1A). The developed setup for fiber enhanced Raman spectroscopy (Figure 2) featured an excitation laser with wavelength λ_exc._ = 785 nm (200 mW output power, Toptica), which was used to avoid fluorescence. The laser light was collimated and cleaned from other laser modes by a notch filter NF (3.0 nm FWHM). The collimated laser beam was focused into a 15 cm long piece of fiber. Within the fiber, the ciprofloxacin molecules were excited by the laser radiation and the Raman scattered light was collected. The backscattered signal was collected with a microscope objective (10×, NA = 0.3) and focused with lens L1 onto the entrance slit of the spectrometer S (Isoplane 320). In order to remove unwanted background originating from the silica cladding structure of the fiber, spatial filtering was performed with lenses L2, L3, and pinhole P. The pinhole size matches the intermediate image of the core, allowing mainly Raman scattered light from the sample to pass. The Raman scattered light from the glass nodes of the cladding structure was thus effectively blocked. Two long pass filters LP1 (0.5% edge steepness) and LP2 (0.5% edge steepness) were introduced to block the Rayleigh scattering and to reduce the stray-light in the spectrometer.

Since the spectral position of the bandgap of the hollow core photonic crystal fiber (HCPCF) is dependent on the refractive index contrast between air/fluid and glass, the transmission band of the fiber is shifted when filled with liquids. A simplified estimation [56] is given by Equation (2), with the refractive index ratios of $N_{0}=n_{glass}/n_{air}$ and $N=n_{glass}/n_{liquid}$.

(2) $$\lambda =\lambda_{0}{\left[\frac{1-N^{-2}}{1-N_{0}^{-2}}\right]}^{\frac{1}{2}}.$$

**Figure 3:**
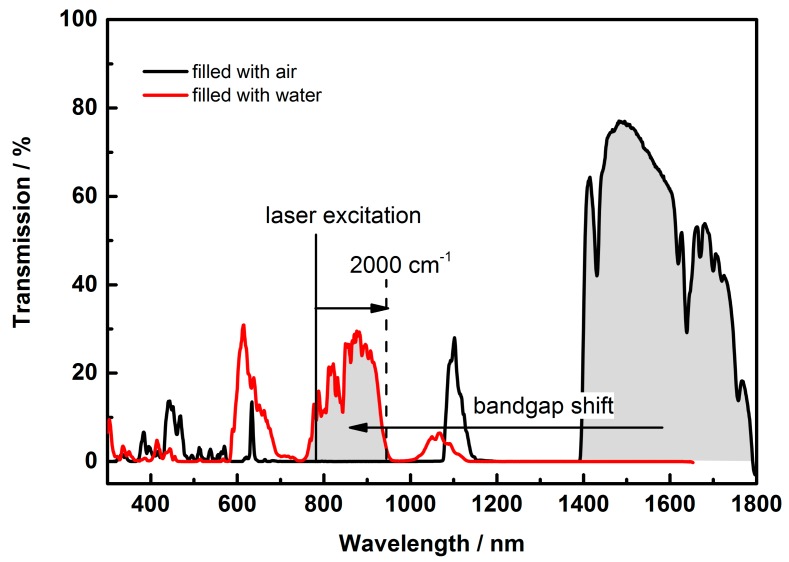
Transmission spectrum of the photonic bandgap fiber. The center of the bandgap of the fiber is dependent on the refractive index difference between the filling material in the central hole (air or liquid solutions) and the structure material (silica). The center of the transmission spectrum of the fiber is shifted from 1550 nm to 865 nm if the fiber is filled with aqueous solutions.

The approximated shift of the center of the transmission band of the hollow fiber from 1550 nm to 865 nm is confirmed in the transmission measurement (Figure 3). Within the wavelength range from 750 nm to 950 nm, a Raman spectrum up to 2000 cm^−1^ can be guided (excitation laser with wavelength λ_exc._ = 785 nm). The spectral window is large enough to compensate for minor changes in the refractive index of the liquid. For liquids with a refractive index different from water (e.g., blood serum), the laser has to be matched to the shifted bandgap.

**Figure 4:**
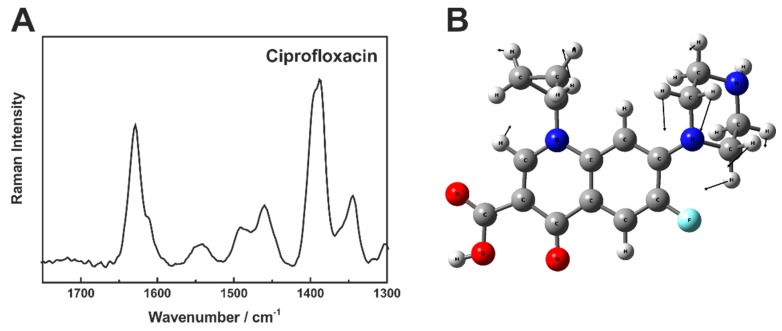
(**A**): Raman spectrum of 12 mM ciprofloxacin in aqueous solution. The spectrum was acquired with the described FERS setup. (**B**): Mode assignment of the Raman peak at 1389 cm^−1^ as bending vibration of ω(CH_2_).

Ciprofloxacin concentrations in the clinically relevant range at 2 µM, 5 µM, 6 µM, 12 µM, and 14 µM were measured with fiber enhanced Raman spectroscopy, as described above. The Raman bands of ciprofloxacin can be clearly identified (Figure 4A). The dominant Raman peak at 1616 cm^−1^ is assigned to the C=C stretching vibration of the quinoline-ring [ν(CC)]. The Raman peak at 1389 cm^−1^ is assigned as CH_2_-wagging vibration of the piperazine and cyclopropyl system [ω(CH_2_)] (Figure 4B) and the peak at 1480 cm^−1^ can be explained as an overlapping of a bending vibration of piperazine [δ(CH_2_)] at 1448 cm^−1^, a bending vibration of cyclopropyl [δ(CH_2_)] at 1484 cm^−1^, and a combination of a νC=C stretching of quinolone ring with a bending vibration of the piperazine system at 1498 cm^−1^.

**Figure 5:**
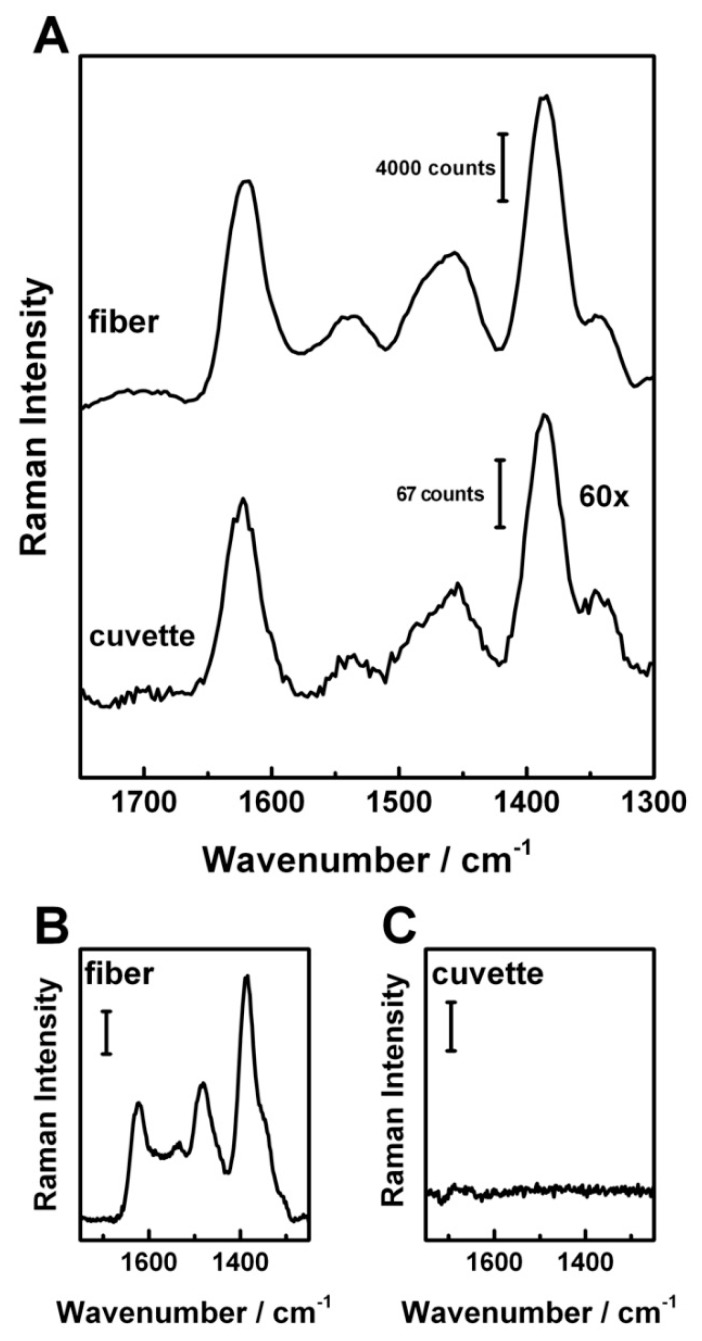
Comparison of the fiber enhanced Raman spectrum and cuvette measurements for a 15 mM ciprofloxacin solution with same setup parameters. An intensity enhancement factor of 60 was achieved with FERS in comparison to the cuvette measurement (**A**). Comparison of an 18 µM ciprofloxacin solution measured with the FERS setup (**B**) and in a cuvette (**C**). The FERS signal is clearly visible (scale bar 1000 counts), while no Raman peak could be detected in the cuvette measurement (scale bar 100 counts), using the same laser power and integration time and after water background subtraction).

In comparison with the conventional cuvette setup, a signal intensity enhancement factor of about 60 was achieved with help of the sensor fiber (Figure 5A). The fiber enhancement results in a better signal to noise ratio and therefore enables the quantification of very low concentrations, down to 2 µM.

The 18 µM ciprofloxacin solution could only be detected with help of FERS (Figure 5B) and was not detectable without fiber enhancement (Figure 5C).

**Figure 6:**
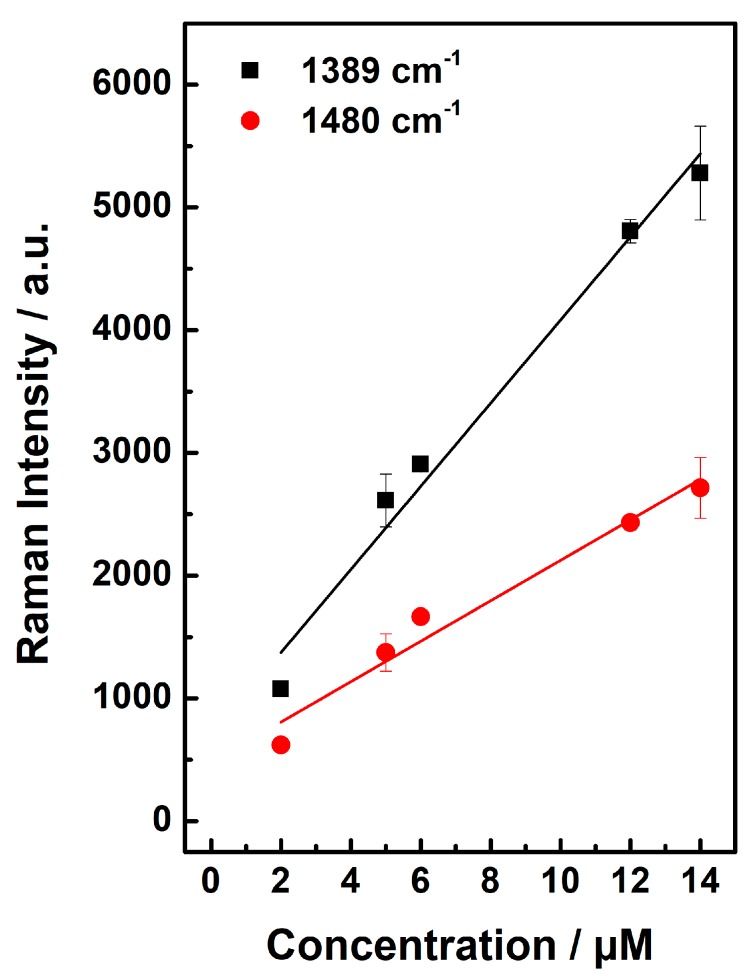
Calibration curve for ciprofloxacin: Very good linearity between Raman intensity and ciprofloxacin concentration was achieved for the Raman peaks at 1389 cm^−1^ (r^2^ = 0.99) and 1480 cm^−1^ (r^2^ = 0.98).

The height of the Raman peaks at 1389 cm^−1^ and 1480 cm^−1^ showed very good linearity (r^2^ = 0.99 at 1389 cm^−1^ and r^2^ = 0.98 at 1480 cm^−1^) with the ciprofloxacin concentration. Thus, a stable calibration could be achieved (Figure 6). All SNR values are summarized in Table 1.

## 4. Conclusions and Outlook

The presented FERS setup enabled the detection of low concentrations: Using a sensor fiber, an enhancement factor of 60 was achieved and ciprofloxacin could be detected in clinically relevant concentrations down to 2 µM. This is especially important in the case of newborn babies suffering from sepsis (6 to 8 µM [5]). Compared to a conventional confocal setup using a cuvette as the sample container, a strong signal enhancement was achieved. The Raman bands of ciprofloxacin of an 18 µM solution were not detectable without the fiber enhancement. A stable calibration of the sensor setup could be achieved, based on the very good linearity between ciprofloxacin concentration and Raman peak intensities. Since the FERS technique provided strong enhancement, the integration time could be decreased to 10 times 3 s. The ability for short data acquisition times less than 1 min is valuable for monitoring of antibiotic levels in sepsis treatment, where time is very critical, particularly within the first few hours. The very low sample volume of 0.58 µl required for the FERS measurement is advantageous for a minimally invasive clinical application. Based on our results, FERS would allow the reliable quantification of unknown ciprofloxacin concentrations. Further tests on body fluids with varying ciprofloxacin levels will approve FERS for individual dose adaption. These tests involve pre-treatment methods like ultrafiltration and centrifugation and a change in excitation wavelength, following the bandgap shift. Ultimately, FERS can allow continuous monitoring of antibiotic levels, such that the dosage can be adjusted correctly in time.

## Figures and Tables

**Table 1:** The table shows the signal to noise ratios (SNR) for different concentrations of ciprofloxacin.

Concentration	SNR	Concentration	SNR
14 µM	20.0	5 µM	9.9
12 µM	18.2	2 µM	4.1
6 µM	11.0		
